# Comparison of hemodynamic and nutritional parameters between older persons practicing regular physical activity, nonsmokers and ex-smokers

**DOI:** 10.1186/1471-2318-10-79

**Published:** 2010-11-01

**Authors:** Cristina O Francisco, Natalia A Ricci, Marcelo N Rebelatto, José R Rebelatto

**Affiliations:** 1 Physiotherapy Department, Federal University of São Carlos (UFSCar), São Carlos, Brazil

## Abstract

**Background:**

Sedentary lifestyle combined with smoking, contributes to the development of a set of chronic diseases and to accelerating the course of aging. The aim of the study was to compare the hemodynamic and nutritional parameters between elderly persons practicing regular physical activity, nonsmokers and ex-smokers.

**Methods:**

The sample was comprised of 40 elderly people practicing regular physical activity for 12 months, divided into a Nonsmoker Group and an Ex-smoker Group. During a year four trimestrial evaluations were performed, in which the hemodynamic (blood pressure, heart rate- HR and VO_2_) and nutritional status (measured by body mass index) data were collected. The paired t-test and t-test for independent samples were applied in the intragroup and intergroup analysis, respectively.

**Results:**

The mean age of the groups was 68.35 years, with the majority of individuals in the Nonsmoker Group being women (n = 15) and the Ex-smoker Group composed of men (n = 11). In both groups the variables studied were within the limits of normality for the age. HR was diminished in the Nonsmoker Group in comparison with the Ex-smoker Group (p = 0.045) between the first and last evaluation. In the intragroup analysis it was verified that after one year of exercise, there was significant reduction in the HR in the Nonsmoker Group (p = 0.002) and a significant increase in VO_2 _for the Ex-smoker Group (p = 0.010). There are no significant differences between the hemodynamic and nutritional conditions in both groups.

**Conclusion:**

In elderly persons practicing regular physical activity, it was observed that the studied variables were maintained over the course of a year, and there was no association with the history of smoking, except for HR and VO_2_.

## Background

A sedentary lifestyle, combined with other risk factors such as smoking, contributes to the development of a set of chronic diseases and to accelerating the course of aging [1]. There is higher prevalence of sedentariness and unsatisfactory quality of life among elderly smokers [1-3]. According to the World Health Organization (WHO), smoking is one of main causes of avoidable death and contributes to the morbidity and disability associated with many chronic illnesses. Among the most common diseases related to smoking are: chronic obstructive pulmonary disease (COPD), lung cancer, myocardial infarction, *diabetes mellitus *type II and arterial hypertension [2,4].

The alterations of aging itself reduce the aerobic capacity and make the elderly more prone to developing arterial hypertension, with smoking being a factor that aggravates this situation [5]. Obesity is another risk factor for developing cardiovascular diseases, and in this case smoking has a protective effect as regards putting on weight, probably because tobacco causes loss of appetite. The association between the smoking habit and mortality due to cardiovascular diseases has been acknowledged, and therefore the protective effect of smoking against putting on weight in no way indicates protection against the risk of cardiovascular diseases [2,4,6].

Modification of lifestyle by adopting healthier habits promotes an improvement in the quality of life and increased life expectancy [5,7]. Physical exercise may help the ex-smoker to improve physical fitness, diminish the risk of chronic diseases, avoid putting on weight, and help to combat the symptoms of abstinence [7,8]. Physical activity also plays an important role in successfully giving up smoking and avoiding a smoking relapse [8]. Epidemiological and laboratory evidences have shown that regular exercise protects against the development and progression of several chronic diseases, and it is considered an important component of a healthy lifestyle [8,9]. When quitting smoking and practicing physical exercise regularly, aerobic capacity may reach the normal values for the age [8].

Considering the effects of exercises on the health of the elderly, the aim of this study was to compare the hemodynamic and nutritional variables of elderly persons practicing regular physical exercise, with different smoking histories.

## Methods

This longitudinal cohort study with an analytical comparative approach was conducted in accordance with the standards demanded by Resolution 196/96 and approved by the Ethics Committee of the Federal University of São Carlos (UFSCar).

This study included individuals aged 60 years or over, of both genders, regularly participating in physical exercise at least three times a week for one year.

A convenience sample of elderly individuals who regularly performed physical exercise was selected among participants of the Adult Revitalization Program. This program has been developed by UFSCar in partnership with the Municipality of São Carlos and seeks to provide the elderly with assistance, by maintaining their physical ability, socialization and improving their quality of life. The physical exercise sessions were held three times a week, lasting 55 minutes for groups of 20 to 30 elderly people. The sessions of physical activity were led by a physical educator and supported by one or two students of physical education (trainees) depending of group size. Physical educators conducted the sessions and the exercise programme included stretching, aerobic conditioning, muscle strength training, coordination, balance, respiratory and relaxation exercises. Those who attended fewer than 75% of the program sessions, or did not participate for 12 consecutive months, and failed to attend the four annual evaluations were excluded. The elderly who met the inclusion criteria were selected (n = 40) and divided into two groups paired by age, and according to smoking history (Nonsmoker Group and Ex-smoker Group), with 20 individuals in each group. All the individuals gave their free informed consent before participating in this study.

In the 12-month period, evaluations were made every three months (total of 4) with tests related to the main variables as regards physical exercise and smoking history. The first evaluation of each elderly subject took place before participation in the Revitalization program.

The nutritional evaluation was obtained by the Body Mass Index (BMI) and the formula used was [weight (kg)/height (m^2^)]. Weight and height were measured on a Filizola^® ^brand anthropometric scale with height measurer.

The hemodynamic data include verification of heart rate (HR) at rest, blood pressure (BP) and maximum oxygen consumption (VO_2 _max.). To measure the HR at rest and BP, the elderly person was seated, with the left forearm supported. HR was checked by radial artery palpation for 15 seconds, and this value was multiplied by four to obtain the HR at rest per minute. The BP was measured with an aneroid sphygmomanometer and a stethoscope, obtaining the systolic blood pressure (SBP) and diastolic blood pressure (DBP).

Maximum VO_2 _was measured indirectly by using the Rockport Walking Test [10,11]. In this test, the participant is required to walk one mile (1609 meters) as quickly as possible. Immediately at the end of the test, the participant's HR and the time spent to complete the mile were recorded. To calculate maximum VO2 the following equation was used: [VO2 max = 132.853 - 0.0769(Weight) - 0.3877(Age) + 6.315(Gender) - 3.2649(Time) - 0.1565(HR)] [10].

The elderly participants were asked about their smoking habits at some time of life and among those who affirmed having smoked, data were collected about the period of time they smoked (years) and time when they stopped smoking (years).

All the evaluations were pre-scheduled, tests were performed on the same day, and lasted approximately one hour. The research team consisted of physiotherapists, physical educators and students (physiotherapy and physical education undergraduates) who were previously trained to apply the tests.

### Data analysis

The evaluation data are shown by means of the developmental results over a period of time; that is, calculating the difference between the evaluation pairs. For the gender comparison the Chi-square test was used. As the Shapiro-Wilks test showed that the data were normally distributed, the parametric paired t-test and t-test for independent samples were applied in the intragroup and intergroup analysis, respectively. In the intergroup analysis, comparison was made between the pair developments (evaluation 1 and 2, evaluation 2 and 3, evaluation 3 and 4, initial and final evaluation). For the intragroup analysis, the comparison between the initial and final evaluation was made. Data analysis was performed by using SPSS Statistical Software ver. 11 (SPSS, Chicago, IL, USA). The significance level for all statistical tests was set at 5% (α = 0.05).

## Results

Forty elderly people who practiced physical exercises for a period of one year were selected, and formed the Nonsmoker Group (n = 20) and the Ex-smoker Group (n = 20). In the Nonsmoker Group there was predominance of women (n = 15), while in the Ex-smoker Group the majority were men (n = 11). There was a statistical trend with regard to the difference between the genders (men versus women) in the formation of the groups (X^2 ^= 3.750 and p = 0.053). As regards age, no statistical difference was found between the groups (p = 1.000). The mean age of the Nonsmoker Group was 68.35 ± 4.93 years and the Ex-smoker Group was 68.35 ± 5.00 years. Minimum age was of 60 years for both the groups and maximum age was 78 and 79 years for the Nonsmoker and Ex-smoker Group, respectively. The Ex-smoker Group smoked for 25.00 ± 15.64 years and quit this habit 20.65 ± 16.45 years ago.

The groups were shown to be similar during the first evaluation for all the variables studied, except for HR at rest (t = 2.683 and p = 0.011). Before starting to practice physical activity, HR at rest of the Nonsmoker Group was shown to be higher than that of the Ex-smoker Group.

The initial data and developmental comparison between the evaluations are shown in Table 1.

**Table 1:** Comparison between Nonsmoker Group and Ex-smoker Group over one year of practicing physical activity

	Nonsmoker Group				Ex-smoker Group			p value
	**Evaluation**		**Development**		**Evaluation**		**Development**	**Intergroup**

		Mean ±	Standard	deviation	Mean ±	Standard	deviation	
	**1**	25.89 ± 4.62			27.75 ± 3.63			0.165
			**1-2**	0.37 ± 0.77			0.50 ± 0.54	0.880
	**2**	25.93 ± 4.43			27.75 ± 3.63			
**BMI**			**2-3**	-0.20 ± 0.54			-0.45 ± 0.53	0.355
	**3**	25.72 ± 4.54			27.72 ± 3.68			
			**3-4**	0.12 ± 0.56			0.59 ± 0.29	0.741
	**4**	25.77 ± 4.67			27.76 ± 3.69			
			**I-F**	0.11 ± 0.25			0.68 ± 0.15	0.762
								
	**1**	78.40 ± 8.55			71.60 ± 7.44			**0.011**
			**1-2**	-3.60 ± 12.44			-1.60 ± 6.54	0.528
	**2**	74.80 ± 11.76			70.00 ± 7.40			
**HR**			**2-3**	1.80 ± 9.84			0.00 ± 5.95	0.488
	**3**	76.60 ± 10.72			70.00 ± 6.81			
			**3-4**	-4.60 ± 10.08			1.40 ± 9.02	0.054
	**4**	72.00 ± 7.89			71.40 ± 10.80			
			**I-F**	-6.40 ± 7.82			-0.20 ± 10.81	**0.045**
								
	**1**	128.25 ± 14.44			136.75 ± 20.02			0.132
			**1-2**	3.25 ± 17.19			-0.25 ± 14.73	0.494
	**2**	131.50 ± 19.54			136.50 ± 22.07			
**SBP**			**2-3**	-5.75 ± 17.42			-1.50 ± 17.55	0.447
	**3**	125.75 ± 15.67			135.00 ± 23.73			
			**3-4**	2.50 ± 15.43			-3.50 ± 13.09	0.193
	**4**	128.25 ± 12.80			131.50 ± 19.27			
			**I-F**	0.00 ± 12.97			-5.25 ± 12.08	0.193
								
	**1**	79.25 ± 11.27			81.00 ± 9.68			0.601
			**1-2**	1.75 ± 9.07			1.50 ± 8.13	0.927
	**2**	81.00 ± 11.19			82.50 ± 8.51			
**DBP**			**2-3**	-4.00 ± 7.54			-2.00 ± 10.05	0.481
	**3**	77.00 ± 9.23			80.50 ± 9.45			
			**3-4**	1.25 ± 9.58			-2.50 ± 11.18	0.262
	**4**	78.25 ± 8.63			78.00 ± 10.56			
			**I-F**	-1.00 ± 12.08			-3.00 ± 13.33	0.585
								
	**1**	20.70 ± 12.11			22.18 ± 6.49			0.640
			**1-2**	0.56 ± 8.19			1.37 ± 5.52	0.721
	**2**	21.26 ± 7.70			23.09 ± 8.97			
**VO_2_**			**2-3**	-0.64 ± 6.70			0.31 ± 6.88	0.683
	**3**	20.17 ± 11.25			24.85 ± 8.99			
			**3-4**	1.47 ± 6.46			2.63 ± 4.19	0.542
	**4**	22.28 ± 9.07			26.39 ± 7.65			
			**I-F**	1.57 ± 7.77			3.37 ± 7.04	0.447

I-F = initial and final evaluation

In the developmental comparison between the first and last evaluation a difference in HR at rest was verified, with a reduction in the Nonsmoker Group when compared with the Ex-smoker Group (t = 2.076 and p = 0.045). For all the other data, no difference was found between the groups in the developmental evaluation over a period of one year.

In the intragroup analysis it was found that after one year in the program, there was a significant reduction in the HR at rest in the Nonsmoker Group (t = 3.655 and p = 0.002) and a significant increase in VO_2 _in the Ex-smoker Group (t = -2.910 and p = 0.010). The other data showed no statistically significant difference (p > 0.05) between the initial and final evaluation within group.

## Discussion

Physical exercise is considered important to promote health, maintain physical capacity, as well as to help combat smoking relapse [1,9,12]. Arterial hypertension is common among the elderly due to the increase in vascular resistance secondary to aging, and physical exercises are recommended to reduce blood pressure [13]. With the aging process there is a decline in cardiovascular performance with a reduction in maximum HR and VO_2 _[14]. There is high association between obesity and arterial hypertension [15] and BMI values above normality are related to the increase in mortality due to cardiovascular diseases [16,17]. The trend towards increasing obesity occurs parallel to the reduction in practicing physical activity [18]. Chronic diseases become more frequent with advancing age and the smoking habit may be a potentiating factor for clinical complications [2,4]. Regular practice of physical exercises has frequently been recommended to treat arterial hypertension, obesity and to quit smoking [13,19].

The training at lower intensity increases fitness levels, and has a favorable effect on variables such as weight, body composition and some blood lipids. Even lower intensity exercise results in improvements in cardiovascular risk factors, especially as regards reducing BP [20].

In this study it was observed that the mean BMI of the Ex-smoker Group was higher than that of the Nonsmoker Group (27.75 kg/m^2 ^and 25.89 kg/m^2 ^respectively), but with no statistical difference (t = -1.416 and p = 0.165). From the cut-off points recommended by the WHO for the adult and elderly population it was found that both groups were within the overweight range (25.0 kg/m^2 ^≤ BMI < 30.0 kg/m^2^). Due to alterations in body composition as a result of aging, the overweight cut-off point for elderly people is BMI > 27 kg/m^2 ^[21]. Based on this classification, the Nonsmoker Group would be within the adequate BMI range and the Ex-smoker Group would be in the threshold range between adequate and overweight. The smoking habit leads to an increase in the basal metabolic rate. With the interruption of smoking there is an increase in weight [22]. Regular physical exercise contributes to maintaining weight, and obtaining lower fat percentage and BMI indexes [13,16].

Epidemiological and cohort studies have shown a strong association between obesity and physical inactivity, and the benefits of physical exercise in body weight control are achieved with low, moderate or high intensity exercises [15]. Silva and Lima [23] verified that physical exercise practiced by elderly people provided significant alterations in BMI, in addition to improving cardiac efficiency by reducing HR at rest by up to 10 bpm after 10 weeks of activities.

There was a difference between the groups with regard to HR in the evaluation before they began practicing exercises, with higher values in the Nonsmoker Group when compared with the Ex-smoker Group. After performing physical exercises, it is common for the HR at rest to be lower, due to a reduction in the cardiac sympathetic tonus [14]. These data corroborate the findings of the intragroup analysis, in which it was found that after one year of practicing physical activity, there was significant reduction in HR in the Nonsmoker Group. The Ex-smoker Group was shown to maintain the HR values, indicating that they had a good level of fitness, and in the last evaluation both groups showed similar values.

Smoking is related to the pathogenesis of cardiovascular diseases such as arterial hypertension [2,4]. In this study, the BP values were shown to be within the standards of normality, however, the Ex-smoker Group showed higher values than the Nonsmoker group, but with no statistical difference. Maintaining these values within the standards may be attributed to regular exercise. Mediano et al [24] verified a reduction of clinical BP in individuals with hypertension after a period of approximately ten months of moderate aerobic physical exercise. Thus, regular and adequate physical exercise must be recommended to maintain and reduce blood pressure.

Maximum VO2 represents the capacity of the body to extract oxygen during a vigorous demand resulting from exercise and it is an important predictor of physical independence and risk of mortality. With aging there is a decline of 10% in maximum VO_2 _per decade after 40 years of age. Physical exercise is capable of reducing this decline to 6% per decade. Due to biological differences between the genders, the normal values for maximum VO_2 _of women are lower than those of men. Middle-aged and elderly women have difficulty in avoiding the decline of VO_2 _due to the reduction in the level of estrogen resulting from aging, their smaller muscle mass, lower hemoglobin and blood volume [25]. By means of VO_2 _and HR, Stevenson and Topp [26] observed significant improvement in the aerobic capacity of elderly people, with low intensity physical training. In the intragroup analysis, this study verified a significant increase in VO_2 _for the Ex-smoker Group after one year of activities in the program. The Nonsmoker Group did not show the same performance and this difference is probably due to the difference in the composition of the group as regards gender. According to the *American Heart Association *(AHA) [27] the level of physical fitness of women between the ages of 60 and 69, based on maximum VO2, is from 13 to 17 ml(kg*min) for weak fitness, 18 to 23 ml(kg*min) for regular fitness and 24 to 34 ml(kg*min) for good fitness; for men from 16 to 22 ml(kg*min) shows weak fitness, 23 to 30 ml(kg*min) regular fitness and 31 to 40 ml(kg*min) good fitness (26). The Nonsmoker Group showed VO2 of 20.70 and 22.28 ml (kg*min) for the first and last evaluation, respectively, being within the regular fitness range when considering the levels for women. Whereas the Ex-smoker Group, considering the level of physical fitness of men, began with weak fitness (22.18 ml (kg*min) and showed regular fitness in the last evaluation (26.39 ml (kg*min). It is worth mentioning that in this research, the VO2 was measured indirectly, which may be related to the differences observed.

Even in the Ex-smoker Group, the hemodinamic variables were within the range of normality, which may be a result of the long period of time since they had stopped smoking. Quitting smoking brings immediate advantages in the medium/long term, the earlier the habit is quit the better. Quitting smoking before the age of 50 diminishes the risk of death in the next 15 years by 50%. After 15 years of abstinence, the risk of cardiovascular disease is equal to that of a non-smoker of the same age and gender [28].

The process of senescence predicts functional losses. The values of hemodynamic and nutritional variables observed in this study, show that regular and long term practice of physical exercises is a preventive strategy capable of maintaining the health status of the elderly population [29] even among those with a history of smoking [12]. Regular physical exercises and quitting smoking may protect individuals against the development and progression of many chronic diseases, and it is considered an important component of a healthy lifestyle [8]. Physical activities with groups of 20 to 30 healthy elderly can be, beneficial and effective in improving physical fitness, so this is a low cost alternative to public health intervention [30].

This study has several limitations. First, the participants were selected as a sample of convenience without a control group. Therefore, one could not infer that the changes observed were in relation to the exercise or due to the aging process, however, due to the study design, it would not be feasible to allow a control group to abstain from physical activity, considering the innumerable indications of the benefits derived from it. Second, the lack of control of variables such as the use of medications, control of exercises intensity and existent diseases in the sample represents other limitation. Probably more vigorous exercises might have caused more significant differences in the studied variables, but the long-term and low intensity programs focus their goals on quality of life and functional independence of the elderly. Stenvenson and Topp [26] found that there was no difference in the outcome of physical activity findings between moderate and low intensity exercise training in the elderly community.

In this study, the exercises was not specifically designed for quitting smoking. Costa et al [31] observed that the multi-professional approach to the treatment of smoking prevents and identifies tobacco-related diseases, helps in basic disease control, promotes changes of habit (food and physical activity) and improves the cardio-respiratory condition. Furthermore, this approach increases adhesion to the program of nicotine abstinence in the long term.

Quitting smoking at any age reduces the risk of death and improves the general health condition [32-35]. Moderately intense exercises have positive effects on the symptoms of giving up smoking [7]. Studies have shown that exercise reduces the desire for nicotine, restlessness, sleeping disturbances, tension and stress and promotes improvement in self-esteem, and contributes to preventing relapse [8,12]. Studies that associate a history of smoking and the importance of adopting healthier habits for improving the quality of life and increasing life expectancy of the elderly are scarce.

## Conclusion

This study showed that exercise is beneficial to reduction in HR and increase in VO_2_. Although, among those without a history of smoking, we found a better improvement in HR, while in ex smokers, it was more efficient affecting VO_2_. These findings reinforce the importance of quitting smoking and show that after a long period of time having stopped smoking, associated with regular physical exercise, the deleterious effects of the habit have a low effect on the hemodynamic and nutritional variables of elderly people.

## Competing interests

The authors declare that they have no competing interests.

## Authors' contributions

COF and NAR were responsible for the design, data acquisition and were involved in drafting the manuscript; MNR worked on data acquisition; JRR was responsible for the conception of this research, and critical review of the manuscript. All authors contributed to and take full responsibility for the article and gave their final approval of the version to be published.

## Pre-publication history

The pre-publication history for this paper can be accessed here:

http://www.biomedcentral.com/1471-2318/10/79/prepub
